# Formalin fumigation and steaming of various composts differentially influence the nutrient release, growth and yield of muskmelon (*Cucumis melo* L.)

**DOI:** 10.1038/s41598-021-99692-0

**Published:** 2021-10-26

**Authors:** Ghulam Mustafa, Muhammad Arif Ali, Donald L. Smith, Sajid Masood, Muhammad Farooq Qayyum, Niaz Ahmed, Ateeq ur Rehman, Shakeel Ahmad, Sajjad Hussain, Muhammad Arshad, Summia Muneer, Aqib Hassan Ali Khan, Shah Fahad, Rahul Datta, Mazhar Iqbal, Timothy D. Schwinghamer

**Affiliations:** 1 Department of Soil Science, Faculty of Agricultural Sciences and Technology, Bahauddin Zakariya University, Multan, 60800 Pakistan; 2 Department of Plant Sciences, Faculty of Agricultural and Environmental Sciences, McGill University, Macdonald Campus 21111, Lakeshore Road, Ste-Anne-de-Bellevue, QC H9X 3V9 Canada; 3 State Key Laboratory of Soil and Sustainable Agriculture, Institute of Soil Science, Chinese Academy of Sciences, Nanjing, 210008 China; 4 Department of Plant Pathology, Faculty of Agricultural Sciences and Technology, Bahauddin Zakariya University, Multan, 60800 Pakistan; 5 Department of Agronomy, Faculty of Agricultural Sciences and Technology, Bahauddin Zakariya University, Multan, 60800 Pakistan; 6 Department of Horticulture, Faculty of Agricultural Sciences and Technology, Bahauddin Zakariya University, Multan, 60800 Pakistan; 7 Institute of Environmental Sciences and Engineering, School of Civil and Environmental Engineering, National University of Sciences and Technology (NUST), Islamabad, 44000 Pakistan; 8 Institute of Plant Breeding and Biotechnology, Muhammad Nawaz Sharif, University of Agriculture, Multan, Pakistan; 9 Department of Earth and Environmental Sciences, Bahria University, Karachi Campus, Karachi, 75260 Pakistan; 10 Hainan Key Laboratory for Sustainable Utilization of Tropical Bioresource, College of Tropical Crops, Hainan University, Haikou, 570228 China; 11 Department of Geology and Pedology, Faculty of Forestry and Wood Technology, Mendel University in Brno, Zemedelska1, 61300 Brno, Czech Republic; 12 Department of Environmental Sciences, Faculty of Biological Sciences, Quaid-i-Azam University, Islamabad, 45320 Pakistan

**Keywords:** Plant sciences, Environmental sciences

## Abstract

Nutrient disorder and presence of disease-causing agents in soilless media negatively influence the growth of muskmelon. To combat these issues, use of environmentally-friendly sanitation techniques is crucial for increased crop productivity. The study was conducted under greenhouse and field conditions to investigate the effect of two different sanitation techniques: steaming and formalin fumigation on various media’s characteristics and their impact on muskmelon yield. Media: jantar, guar, wheat straw and rice hull and peat moss of 10% air-filled porosity and sanitized with formalin and steaming. Steaming of guar, jantar, and wheat straw increased the phosphorus (P) and potassium (K) concentrations by 13.80–14.86% and 6.22–8.45% over formalin fumigation. Likewise, P and K concentrations in muskmelon were higher under steaming. Steaming significantly inhibited the survival of *Fusarium* wilt sp. *melonis*, root knot nematode sp. *meloidogyne* and nitrifying bacteria in media than formalin fumigation. In conclusion, steaming decreased the prevalence of nitrifying bacteria and pathogens which thus improved the NO_3_^−^–N:NH_4_^+^–N ratios, P and K nutritional balance both in the media and muskmelon transplants. Hence, steaming as an environment-friendly approach is recommended for soilless media. Further, optimization of steaming for various composts with different crops needs to be investigated with steaming teachnique.

## Introduction

Plant based-media have a great potential as an alternative to peat moss. It may limit the use of soils and reduce the fertilizer cost for transplant growth^1^. Farmers can also earn an additional income by marketing their own potting media^2^. However, characteristics i.e. air-filled porosity (AFP) of the media are of major concern. Generally, AFP ranges 10–30% in the media based on different particles sizes^3–6^.

After establishing AFP, sanitation techniques or approaches are mandatory for maintaining of the composition and nutrient balance in potting media. Sanitation has been known to alter the composition of composts and may better respond to the plant’s specific requirements and increased nutrient release, thereby improving the growth, yield, and food quality^7,8^. In addition, soil sanitation with methyl bromide, chloropicrin and steaming in pot culture improved the nitrogen dynamics and beneficial microbial biomass^9^ and reduced the pest attack^10^. Similarly, sanitation of soil using fumigants has been implicated with reduced pathogens, replants disease and enhanced the peach growth^11^.

Chemicals may also reduce the severity of both soil and seed-borne diseases^12^. For instance, soil fumigation with metam sodium decreased the potato scab by 98%^13^. Ammonia gas fumigation in the cucumber field suppressed the harmful plant pathogen causing *Fusarium* wilt disease and enhanced the yield^14^. Likewise, dimethyl sulphide was an effective soil fumigant against nematodes^15^. Other than chemicals, steam disinfection^16^, soil solarization^17^, and biochar application^18^ are effective. Disinfection techniques kills nematodes and pathogens, however may harm beneficial microbes and have negative impacts on the plant growth and development^19^. In addition, peat alternatives and sanitation techniques should fulfill media’s health requirements^20^.

Agricultural wastes must be considered as an attractive source for making value-added products in order to address food security challenge^12,21^. In this regard, guar, jantar, wheat straw and rice hull are abundant agricultural wastes that can be utilized and processed through composting. Guar and jantar being leguminous in nature, have low nutrient requirements for their production, commercially produced and may be utilized for N source of fertilization^22^.

Muskmelon is an important vegetable crop from *Cucurbitaceae* family cultivated throughout the world^23,24^. Additionally, it is a short duration crop and can respond quickly to the nutrient supply while raising muskmelon seedlings in the composts^25,26^. There is a need to ascertain which type of sanitation is suitable for particular media and growth of transplants. Although different sanitation methods of the media have been reported, very little is known about surviving ability of pathogens, nitrifying bacteria, nitrification inhibition and nutrient mineralization in media-plant systems. We hypothesized that steam sanitation of the media not only kills the population of nitrifying bacteria and disease-causing pathogens but also balances and increases nutrients availability to the plants than formalin fumigation. As a result, competition between the plants and pathogens is strongly inhibited for nutrient uptake. The present study was designed to assess; (1) surviving ability of nitrifying bacteria and other pathogens under the influence of steaming and formalin fumigation of the media, (2) characterization of the composts under the influence of specific sanitation technique for their nutrient release potential and improving the growth and yield of muskmelon.

## Results

### Germination, mortality and muskmelon yield

**Figure 1:**
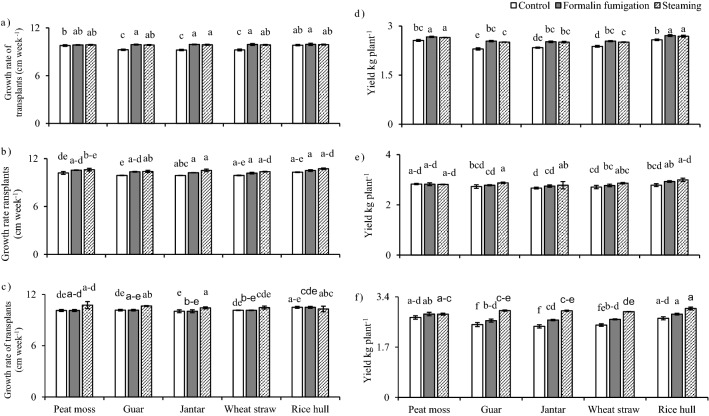
Effect of composts and sanitation techniques on: (**a**) growth rate (cm week^−1^) for site 1; (**b**) growth rate (cm week^−1^) for site 2; (**c**) Growth rate (cm week^−1^) for site 3; (**d**) yield (kg plant^−1^) for site 1; (**e**) yield (kg plant^−1^) for site 2, (**f**) yield (kg plant^−1^) for site 3 of muskmelon. All the values are means ± S.E of three replicates, whereas case letters indicate significant differences among the treatments at *p* ≤ 0.05 level.

Interactive effects of media and sanitation techniques were significant for plant growth traits like seed germination, root length, leaf chlorophyll contents, number of leaves, leaf area, stem thickness and shoot fresh weights (Table 1). The generalized ANOVA exhibited significance (F-value) for root length (29.37), no. of leaves per seedling (468.69), leaf area (6.94) and shoot fresh weight (3.49) at *p* ≤ 0.05, 0.01 and 0.001 levels. Overall, steaming of composts performed better for growth rates and yield among sanitation treatments followed by formalin fumigation and unsanitized control (lowest yield). Rice hull showed the best growth and yield among composts (Fig. 1a–c).

**Table 1:** Estimates of least square means ± standard errors calculated on account of natural log (on the scale of inference) with their exponential means (on the scale of measurement) for the determination of experimental variables and their probabilities of germination.

Compost	Sanitation	Germination (%)		Root length (cm)		Shoot height (cm)		TNL/seedling*	Leaf area (cm^2^)		Chlorophyll contents (SPAD value)	Fresh weight (g)	
		Est. ± SE* $Ln\left( {\frac{\pi }{1 - \pi }} \right)$	Prob.* π × 100 ± *se*	Est. ± *SE*	Exp.* (cm)	Est. ± *SE*	Exp. (cm)	Est. ± *SE*	Est. ± *SE*	Exp. (cm^2^)		Est ± *SE*	Exp. (g)
Peat moss	Control	2.02 ± 0.05a	88.33 ± 0.52	1.90 ± 0.01e	6.67	1.89 ± 0.03d	6.60	4.00 ± 0.00b	3.60 ± 0.03cd	36.70	38.93 ± 0.83 f.	1.43 ± 0.03 d	4.17
	Formalin	2.19 ± 0.05a	89.97 ± 0.48	2.01 ± 0.02d	7.47	1.95 ± 0.02bcd	7.00	4.00 ± 0.00b	3.64 ± 0.03bc	38.16	49.63 ± 1.36cde	1.52 ± 0.02ab	4.57
	Steam	2.19 ± 0.05a	89.97 ± 0.48	2.02 ± 0.01d	7.43	1.99 ± 0.02ab	7.33	4.00 ± 0.00b	3.66 ± 0.04ab	38.87	53.67 ± 0.88bc	1.48 ± 0.02bc	4.41
Guar	Control	1.61 ± 0.04b	83.33 ± 0.61	1.87 ± 0.02e	6.47	1.90 ± 0.02d	6.70	3.66 ± 0.01c	3.58 ± 0.03d	35.83	42.63 ± 0.97f	1.44 ± 0.02cd	4.21
	Formalin	2.02 ± 0.05a	88.33 ± 0.52	2.06 ± 0.03abc	7.83	1.98 ± 0.01abc	7.23	4.00 ± 0.00b	3.64 ± 0.02bc	38.03	55.37 ± 1.03bc	1.53 ± 0.03ab	4.59
	Steam	2.02 ± 0.05a	88.33 ± 0.52	2.06 ± 0.01abc	7.83	1.95 ± 0.01bcd	7.00	4.00 ± 0.00b	3.66 ± 0.04ab	38.90	57.13 ± 4.27b	1.53 ± 0.02ab	4.61
Jantar	Control	1.61 ± 0.04b	83.33 ± 0.61	1.90 ± 0.01e	6.70	1.88 ± 0.01d	6.53	3.69 ± 0.01c	3.59 ± 0.03d	36.17	40.27 ± 1.51f	1.43 ± 0.03d	4.17
	Formalin	2.19 ± 0.05a	89.97 ± 0.48	2.03 ± 0.01bcd	7.63	1.94 ± 0.02bcd	6.97	4.00 ± 0.00b	3.68 ± 0.04ab	39.47	50.89 ± 1.57bcd	1.55 ± 0.02a	4.71
	Steam	2.19 ± 0.05a	89.97 ± 0.48	1.99 ± 0.02d	7.33	1.93 ± 0.01bcd	6.90	4.00 ± 0.00b	3.66 ± 0.02ab	38.77	55.72 ± 9.57bc	1.52 ± 0.03ab	4.59
Wheat straw	Control	1.61 ± 0.04b	83.34 ± 0.61	1.91 ± 0.02e	6.73	1.92 ± 0.01cd	6.83	3.69 ± 0.01c	3.58 ± 0.01d	35.80	44.03 ± 1.39ef	1.43 ± 0.02d	4.18
	Formalin	2.03 ± 0.05a	88.36 ± 0.52	2.01 ± 0.01d	7.47	2.02 ± 0.02a	7.57	4.00 ± 0.00b	3.64 ± 0.02bc	38.10	51.93 ± 0.97bcd	1.52 ± 0.01ab	4.59
	Steam	2.03 ± 0.05a	88.4 ± 0.52	2.02 ± 0.03cd	7.53	2.00 ± 0.03ab	7.40	4.00 ± 0.00b	3.66 ± 0.03ab	38.87	52.93 ± 7.28bc	1.53 ± 0.03ab	4.64
Rice hull	Control	2.02 ± 0.05a	88.31 ± 0.52	2.01 ± 0.03d	7.47	1.99 ± 0.02ab	7.33	4.33 ± 0.01a	3.58 ± 0.02d	35.93	45.37 ± 1.08def	1.43 ± 0.02d	4.17
	Formalin	2.40 ± 0.06a	91.71 ± 0.44	2.06 ± 0.01ab	7.87	1.99 ± 0.03ab	7.33	4.31 ± 0.01a	3.65 ± 0.02bc	38.33	64.97 ± 1.30a	1.53 ± 0.03ab	4.60
	Steam	2.40 ± 0.06a	91.68 ± 0.44	2.09 ± 0.02a	8.07	1.97 ± 0.01abc	7.19	4.33 ± 0.01a	3.70 ± 0.03a	40.43	70.43 ± 10.31a	1.53 ± 0.03ab	4.59

*Prob.* probabilities, *π* probabilities of germination, *SE* standard error, *Est.* estimates, *Exp.* exponential, *TNL* total number of true leaves.

Means sharing similar letter in a row or in a column are statistically non-significant (*p* ≤ 0.05).

Similar to growth, muskmelon yield was positively influenced by steaming of the composted media (Fig. 1d–f). Muskmelon transplants grown under rice hull compost had maximum yield (3.50–3.25 kg plant^−1^). At all sites, all the media under the influence of steaming increased the yield plant^−1^ than transplants grown in media receiving formalin fumigation.

### Establishing AFP of the composts

Results of the present study revealed that particle sizes greatly influenced the AFPs of the media (Tables S1 and S2). Relative proportions of particles of 2–3.3 mm and < 2 mm were associated with AFPs of guar (R^2^ = 0.94), jantar (R^2^ = 0.83) and wheat straw (R^2^ = 0.91). In the case of rice hull compost, relationship between varying proportions of particles size of 1–2 mm blended with 0.5–1 mm and AFPs was highly significant (R^2^ = 0.88).

### Physicochemical properties of the sanitized media

Electrical conductivity (EC) and pH were not significantly influenced by the sanitation techniques, but remained in the suitable range for the growth of muskmelon (Table S3). Although all the media were of same AFP levels, water holding capacity (WHC) of the composts varied.

### Population of nitrifying bacteria and nitrogen transformation in the media and muskmelon transplants

**Figure 2:**
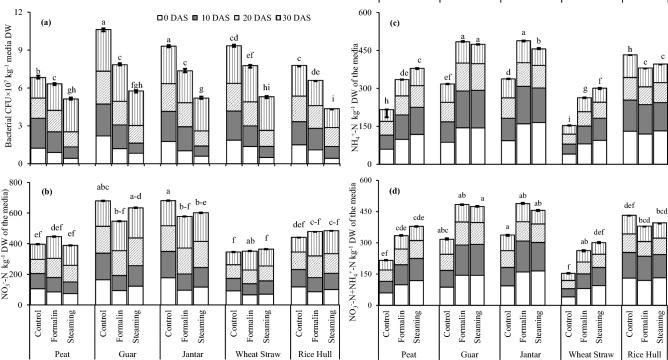
Effect of sanitized media on: (**a**) relative abundance of nitrifying bacteria over the time, (**b**) NO_3_^−^–N mg kg^−1^ media, (**c**) NH_4_^+^–N mg kg^−1^ media and (**d**) NO_3_^−^–N + NH_4_^+^–N mg kg^−1^ media over the time. All the values are means ± S.E of three replicates, whereas case letters indicate significant differences among the treatments at *p* ≤ 0.05 level. All the composts used in greenhouse experiment were established at 10% AFP. DAS = Days were after sanitation.

Sanitation reduced the population of nitrifying bacteria in the media at 0 and 10 d when compared with the control and fumigation, respectively. Nitrifying bacteria got recovered after 20 and 30 d of sanitation, however, population of these bacteria was high 30 d after sanitation of the media (Fig. 2a). In general, steaming sanitation had lower population of nitrifying bacteria than formalin fumigation.

**Figure 3:**
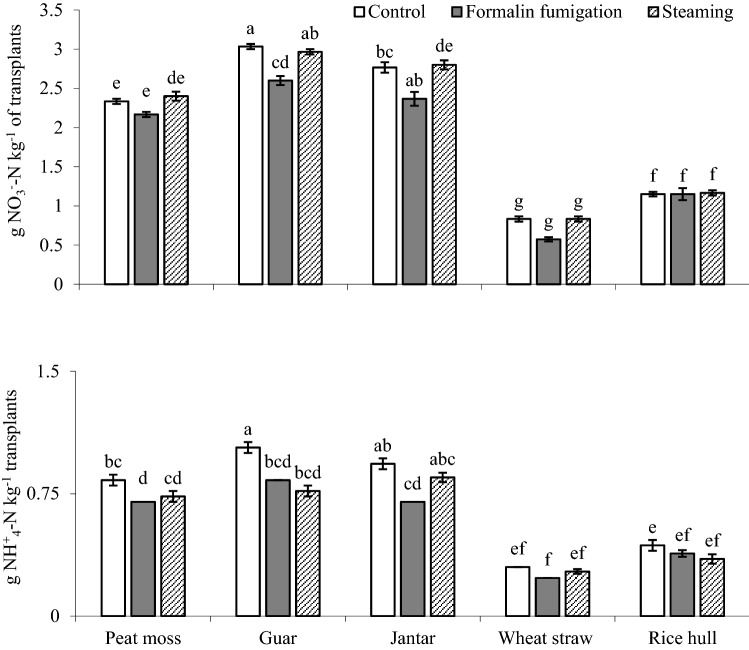
Inorganic-N concentrations: NO_3_^−^–N and NH_4_^+^–N concentrations in muskmelon transplants as influenced by sanitation techniques. All the values are means ± S.E of three replicates, whereas case letters indicate significant differences among the treatments at *p* ≤ 0.05 level.

Nitrate (NO_3_^−^–N) concentrations in all the steam sanitized media except peat moss at 0 and 10 d were increased over formalin fumigated (Fig. 2b). Similarly, ammonium (NH_4_^+^–N) concentrations were increased by steaming of guar and rice hull than formalin fumigation (Fig. 2c). Steaming of the media increased the NO_3_^−^–N concentrations at 20 d and 30 d as compared to formalin fumigation. In contrast to NO_3_^−^–N, NH_4_^+^–N concentrations in the media after 20 d and 30 d of sanitation varied largely among the composts. Moreover, NO_3_^−^–N + NH_4_^+^–N in concentrations in the steam sanitized media were higher than that of formalin-fumigation (Fig. 2d). Nitrate–N and NH_4_^+^–N concentrations were accumulated more in muskmelon transplants which were grown in sanitized media (Fig. 3).

### Nutrient concentrations of the media and muskmelon transplants as influenced by sanitation

**Figure 4:**
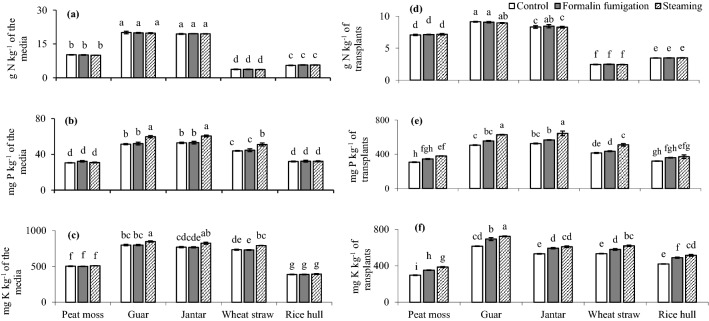
Relative concentrations of nutrients in the media and muskmelon seedlings under different sanitation techniques. Concentrations of various nutrients: (**a**) total N; (**b**) P; (**c**) K in the media; (**d**) N; (**e**) P and (**f**) K in muskmelons transplants have been represented. All the values are means ± S.E of three replicates, whereas case letters indicate significant differences among the treatments at *p* ≤ 0.05 level.

Similar to N-forms, total N concentrations in the steam sanitized media were increased (Fig. 4a). Steam sanitized media increased the P concentrations of guar, jantar and wheat straw by 14.86%, 13.80% and 14.24% greater as compared to the formalin fumigation (Fig. 4b). Similarly, K concentrations in steam sanitized guar, jantar, and wheat straw composted media were increased by 6.22%, 7.54% and 8.45% when compared with the formalin fumigation (Fig. 4c).

Steaming increased the P concentrations in muskmelon transplants grown under peat, guar, jantar, wheat straw, rice hull composted media by 9.97%, 13.03%, 13.59%, 17.25% and 3.25% as compared to formalin fumigation (Fig. 4). Similarly, K concentrations in muskmelon transplants from steam sanitized peat, guar, jantar, wheat straw and rice hull media were 9.62%, 4.32%, 2.61%, 6.82% and 5.26% higher than the transplants received formalin fumigation (Fig. 4).

### Prevalence of root knot nematodes and Fusarium wilt

**Figure 5:**
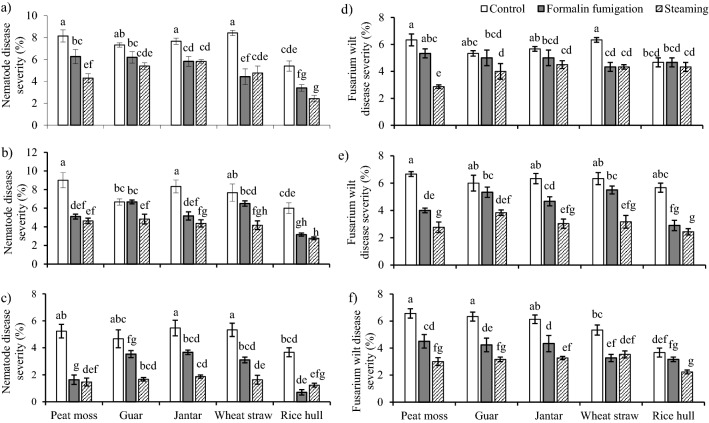
Comparative effect of the composts and sanitation techniques on root knot nematode disease severity (**a**) site 1; (**b**) site 2; (**c**) site 3 and *Fusarium* wilt disease (%), (**d**) site 1; (**e**) site 2; (**f**) site 3. All the values are means ± S.E of three replicates, whereas case letters indicate significant differences among the treatments at *p* ≤ 0.05 level.

Between the two sanitation techniques, steaming significantly reduced the disease severity of root knot nematodes and *Fusarium* wilt (Fig. 5 and Table 3). Moreover, steaming sanitation inhibited the population of disease-causing agents in all the media.

## Discussion

Seed germination and root length are the foremost indicators contributing to plant health and survival. In the present study, higher germination% in the rice hull compost was probably due to non-woody nature of the compost which thus decomposed when sanitized and enhanced seed germination. Rice hull compost provides optimal concentrations of N, P, K and additional supplements like Si, which thus improve the plant growth and development^27^. Moreover, the increase in seed germination and root length of steam sanitized media over their corresponding controls was correlated with the increased nutrient availability^28^. Steaming of the media probably influenced the C:N ratio of the media and increased NO_3_^−^–N availability to muskmelon seedlings. The increased root length and germination rate of muskmlon in the sanitized media suggest that these attributes determine plant survival.

Physical properties of the media are generally influenced by particle sizes and AFP of the composts, which thus contribute to plant growth and development. In the present study, suitable particle sizes were selected for the establishment of 10% AFP. These particles helped in holding moisture as small particles hold more water than the large particles. Moisture retention thus enhanced the germination and sustained the pH and EC in acceptable ranges^28^. Physical properties of the media contributed a lot in enhancing germination of tomato^29^. Since bulk density of the media is dependent on AFPs, thermal conductivity could be influenced differently^30^ and may affect the plant growth. This is well supported by the findings of^31^ who reported a differential convection of heat at various saturation and moisture levels. Penetration of steam through media is easier and higher in comparison to fumigants or dry heating^32^. Additionally, microbial communities are affected by physical properties of the media. In the present study, increased WHC by the composts helped in the promotion of plant growth and development, whereas sanitation of the media influenced the population of nitrifying bacteria and inhibited pathogen attack on muskmelon. In general, nitrifying bacteria contribute significantly to N transformation and nitrification^33,34^. Soil sterilization resulted in re-colonization of healthier microorganisms in the rhizosphere^35^. Recurrent drying and wetting largely affects the microbial biomass^36^. Similarly, warming of temperate forest soil altered the microbial community functioning^37^.

The reduction in nitrifying bacterial community under steaming inhibited the nitrification potential of the media at 0 and 10 d intervals and maintained optimal NO_3_^−^–N:NH_4_^+^–N ratios in muskmelon transplants. Since plant nutrient requirements, especially of N are very low at seedling stage, muskmelon growth in the present study was not affected rather synchronized with the needs of N of transplants at early stages. The increase in NO_3_^−^–N at 20 and 30 d of sanitation were resulted due to recovery of nitrifying bacteria, which thus enhanced the nitrification of NH_4_^+^–N to NO_3_^−^–N. The increase in NO_3_^−^–N at 20 and 30 d of sanitation enhanced the growth of muskmelon seedlings. This implies that crop NO_3_^−^–N requirements were high than NH_4_^+^–N. Previous studies exhibited that NH_4_^+^–N inhibited the maize growth, whereas the elevated levels of NO_3_^−^–N improved the growth of maize. Therefore, suitable ratios of both N-forms are necessary for the optimum plant growth, otherwise NH_4_^+^–N cause toxicity in the plants. In the present study, NO_3_^−^–N:NH_4_^+^–N ratio was closely adjusted to optimal (2:1) during crop growth cycle to reveal the effect of steaming and formalin fumigation. However, best plants growth was observed at 75:25 ratio of NO_3_^−^–N to NH_4_^+^–N^38^. These differences possibly resulted due to variations in crop genotypes, growth conditions, and growth medium.

The increase in nutrient uptake by muskmelon seedlings in sanitized media was resulted due to mineralization potential and WHC of the media. Since root lengths of muskmelon seedlings were increased in sanitized media, these interacted with the mineralized nutrient pool and absorbed water. Higher acquisition of K by muskmelon seedlings was achieved possibly due to competition between K and NH_4_^+^–N at root interface. This has been reported that K uptake was increased with the increase in NH_4_^+^–N^39^. In view of competition mechanism, anion like NO_3_^−^–N favored in PO_4_^3−^ accumulation in muskmelon seedlings. In the present study, suitable NO_3_^−^–N:NH_4_^+^–N ratio synergistically contributed to the uptake of P. In another study, suitable NO_3_^−^–N:NH_4_^+^–N ratio enhanced P uptake by maize^40^. In addition, N:P ratios influence the fungi, bacterial colonization, availability and uptake of nutrients^41^.

In spite of this, transplant shock under field conditions is obvious. Preventing transplants from shock, pathogenic attack and soil borne disease are major challenges in recent years. In the present study, media acted as bio-fumigants which reduced the widely distributed plants diseases like *Fusarium wilt* and pathogens like nematodes. Moreover, steaming of the media eradicated root nematode disease and *Fusarium wilt* more than formalin fumigation possibly due to increased NO_3_^−^–N uptake which in turn provided resistance against pathogenic attack and transplant shock. Several media alternatives like vinegar residue and spent coffee increased the resistance in plants against *Fusarium wilt*^42^ and enhanced the growth of basil and tomato^43^. In the present study, sanitation-induced effects on transplants growth persisted after transplantation and improved the growth of transplants under field conditions. The post-transplantation improvement in growth rate and yield of muskmelons was subjected to beneficial interaction effect of sanitation with guar and jantar and wheat straw media. Microbiome changes in rhizosphere led to decrease in root knot nematodes^44^. Fumigation with ammonium biocarbonate and organic fertilizer, suppressed the *Fusarium wilt* to 12% in watermelon and enhanced the yield^45^. Treating of plug trays at 65 °C for 60 min manifested in the reduction of phytophathora^46^. In the present study, steaming provided resistance in the muskmelon transplants against root knot nematodes and Fusarium wilt, thereby improved the growth and yield.

## Conclusions

Steaming sanitation decreased the prevalence of nitrifying bacteria and inhibited nitrification in steaming thus improved the NO_3_^−^–N:NH_4_^+^–N ratios, P and K nutritional balance both in the media and muskmelon transplants than formalin fumigation. Additionally, steaming reduced pathogens and diseases in plants thus improved muskmelon growth and yield more than formalin fumigation. Based on our findings, steaming being a non-chemical and environment-friendly approach is recommended for soilless media. Further, optimization of steaming for various composts to use as media for various crops needs to be investigated with steaming technique.

## Material and methods

### Experimentation, climatic conditions and determination of disease severity

Muskmelon (*Cucumis melo* L. cv. Melon) nursery was raised in plug trays which contained all the media either sanitized or not. There were different plug trays for each of the media: wheat straw, guar, jantar, and rice hull and sanitation techniques: steaming and formalin. One seed in each hole of the plug trays was sown for 30 d. Germination percentage, mortality, seedling height, root length, number of true leaves per seedling and seedlings fresh weights were recorded.

Afterwards, seedlings were transplanted in three different fields located in Tehsil Mailsi, District Vehari, Punajb, Pakistan. Before transplantation, surface soil samples (0–15 cm depth) were collected for physico-chemical analysis (Table 2). Soils were prepared by conventional tillage practices and chemical fertilizers i.e. NPK were applied once at the time sowing from their sources: urea, di-ammonium phosphate (DAP), and sulphate of potash (SOP) at the rate of 200 kg N, 150 kg P_2_O_5_, and 110 kg K_2_O ha^−1^, respectively. The dimensions of the beds and furrows were: 150 cm wide × 60 cm wide, whereas 14,680 transplants ha^−1^ were maintained. All the cultural and management practices were implemented throughout the experiment.

**Table 2:** Soil physico-chemical properties of the experimental sites.

Site	Address	Latitudes longitudes	Depth (cm)	Texture	pH	EC (dS m^−1^)	SAR	SOM (%)	N (%)	P (mg kg^−1^ soil)	K (mg kg^−1^ soil)
Site 1	Ghallu, Mailsi	29.89° N 72.08° E	15	Sandy loam	8.07 ± 0.32	0.86 ± 0.02	3.56 ± 0.12	0.76 ± 0.03	0.04 ± 0.002	6.41 ± 0.23	83 ± 3.92
Site 2	Lakhokha, Mailsi	29.88° N 72.06° E	15	Loam	8.23 ± 0.29	1.03 ± 0.03	2.84 ± 0.11	0.91 ± 0.04	0.05 ± 0.002	7.93 ± 0.33	167 ± 7.42
Site 3	Marri Mitru, Mailsi	29.80° N 72.17° E	15	Clay loam	8.34 ± 0.18	0.97 ± 0.04	3.11 ± 0.13	0.87 ± 0.04	0.05 ± 0.002	7.17 ± 0.27	173 ± 8.01

All the values means ± S.E of three replicates.

Plants received natural sunlight and other climatic conditions of the study area are: mean day/night temperature 32 °C/24 °C with 13 h photoperiod and 51–52% relative humidity.

Muskmelon was grown in the fields till maturity or 105 d, whereas symptomatic plants were randomly selected for the evaluation of nematode disease severity^47^ and *Fusarium* wilt^48^ during whole experiment. Muskmelon yield plant^−1^ was measured at harvesting.

### Preparation of composts and establishing required AFPs and sanitation treatments

Crop residues like wheat straw, jantar, guar and rice hull were subjected to composting by pit method^49^, whereas peat moss (Peltracom N.V., Belgium) was used as a reference material. Particles of sizes > 5 mm, 3.3–5 mm, 2–3.3 mm, < 2 mm, 2–1 mm, 1–0.5 mm and < 0.5 mm were separated passing through sieves of various sizes viz. 0.5, 1, 2, 3.3, and 5.0 mm. The mixes (substrates) were sequentially prepared with different AFPs (Tables S1 and S2). The required AFPs of the substrates were determined employing CEN standard^50^. The substrates of 10% AFPs and peat moss were used as controls and were sanitized with formalin at the rate of 2 ml l^−1^ or steaming at 60 °C for 30 min.

### Determination of physic-chemical properties and fiber contents of the composts

Physico-chemical properties of the potting media like water holding capacity (WHC), bulk density and shrinkage percentage were determined^50^ (Table S3). Fiber contents of the substrates were separated by manual shaking in a container and their volume was quantified.

### Determination root knot nematode sp. meloidogyne and Fusarium wilt sp. melonis population

Number of second stage juveniles (J2s) of root knot nematodes (*Meloidogne* sp.) were determined using hemocytometer and expressed as number of juveniles (J2s) root knot nematodes kg^−1^ of the media^51^ (Table 3). Similarly, spore farming units of *F. oxysporium* sp. *melonis* were quantified in the suspensions and serial dilutions^52^ (Table 3).

**Table 3:** Effect of composts and sanitation techniques on relative abundance of juveniles of root knot nematode and *Fusarium oxysporium* sp. melonis in the media.

Composts	Sanitation techniques	Nematode J2S stage (10^3^ × kg^−1^ of media)	*Fusarium oxysporium* sp. Melonis (CFUs 10^3^ × kg^−1^ media)
Peat moss	Control	3.33 ± 0.67ab	96.67 ± 3.33c
	Formalin	1.67 ± 0.33b–e	43.33 ± 8.82de
	Streaming	1.33 ± 0.33a–d	33.33 ± 3.33de
Guar	Control	3.00 ± 0.58abc	170.00 ± 5.77ab
	Formalin	2.00 ± 0.00a–d	53.33 ± 14.53d
	Streaming	1.00 ± 0.58a–d	41.67 ± 4.41de
Janter	Control	3.67 ± 0.33a	147.00 ± 3.33b
	Formalin	1.33 ± 0.33cde	40.00 ± 5.77de
	Streaming	0.67 ± 0.33e	36.67 ± 8.82de
Wheat straw	Control	3.00 ± 0.58abc	183.33 ± 8.82a
	Formalin	1.67 ± 0.33b–e	60.00 ± 5.77d
	Streaming	0.67 ± 0.33e	53.33 ± 3.33d
Rice hull	Control	1.33 ± 0.33cde	40.00 ± 5.77de
	Formalin	0.67 ± 0.33de	16.67 ± 3.33e
	Streaming	0.67 ± 0.33e	11.67 ± 1.67e

All the values are means of three replicates, whereas letters exhibit significant differences among the treatments at *p* ≤ 0.05 level.

### Determination of nitrifying bacteria in the composts

Composts samples were collected and brought to the laboratory under sterile conditions after 0, 10, 20 and 30 d (harvest) and abundance of nitrifying bacteria (CFUs) were determined by plating in the medium having chemical composition for all nitrifying bacteria^53^. Briefly, 1 g of the media was mixed with 30 ml using sterile water and serial dilutions of 10^−1^ to 10^−5^ suspensions were spread on agar media.

### Determination of nutrient concentrations in the composts and muskmelon seedlings

For the determination of nutrient concentrations, extracts from the media were collected by shaking 1:5 (w/v) at a speed of 150 rpm for 30 min and used for the measurements of NPK by Kjeldahl apparatus, spectrophotometer and flame photometer, respectively. For the purpose of NO^−3^–N and NH^+^_4_–N concentrations, composts mixes were extracted in 2 M KCl for one hour at a speed of 150 rpm and collected aliquots were used to determine NO^−^_3_–N and NH^+^_4_–N by steam distillation^54^. Likewise, nutrients concentrations like total N, NO^−^_3_–N, NH^+^_4_–N, P, and K in muskmelon transplants were measured after digesting plant materials in acid digestion mixture HNO_3_:HClO_4_ (4:1 v/v).

### Experimental design and statistical analyses

The experiment followed split plot design. In general, there were three replications in each treatment. One-way ANOVA was obtained for statistical evaluation of AFPs of different mixes, whereas two-way ANOVA was used for the interpretation of second stage juveniles (J2s) of disease-causing agents and mortality rate and muskmelon using Statistix 8.1 software. Treatment means of the yield data were compared according to Tukey’s post-hoc test. Generalized ANOVA was performed using SAS PROC Generalized Mixed Model (GLIMMAX) for analyses of the seedlings data. Since the data of variable germination was in the units of percent, modelling was employed by specifying the beta-binomial distribution (DIST = BETA). Data of root length, seedlings height, stem diameter, leaf area, and fresh weight variables were checked for normal distribution (DIST = LOGN). For these variables, standard error was merely usable on the natural log scale, hence estimates of the means are described with 95% confidence limits. Based on the fit statistics, (shifted) *t* distribution was found as suitable for model of the data of number of true leaves per seedling. Moreover, germination% and other seedlings growth attributes were modeled using class variables: compost type, sanitation type, and their interaction (compost x sanitation). Scheffe's adjustments were made for multiple comparisons among the treatments.

### Ethics approval and consent to participate

We all declare that manuscripts reporting studies do not involve any human participants, human data, or human tissue. So, it is not applicable.

### Complies with international, national and/or institutional guidelines

Experimental research and field studies on plants (either cultivated or wild), comply with relevant institutional, national, and international guidelines and legislation.

## Supplementary Information

Supplementary Tables.
