# Supplementary material for: Formalin fumigation and steaming of various composts differentially influence the nutrient release, growth and yield of muskmelon (Cucumis melo L.)
Source: Sci Rep. 2021 Oct 26;11:21057. doi: 10.1038/s41598-021-99692-0 (PMC8548321; doi:10.1038/s41598-021-99692-0)
Supplement: Supplementary file 1 — Supplementary Tables. [file 41598_2021_99692_MOESM1_ESM.docx]

**Formalin fumigation and steaming of various composts differentially influence the nutrient release, growth and yield of** **muskmelon (*Cucumis melo* L.)**

Ghulam Mustafa^1*^, Muhammad Arif Ali^1*^, Donald L. Smith^2^, Sajid Masood^3^, Muhammad Farooq Qayyum^1^_,_ Niaz Ahmed^1^, Ateeq ur Rehman^4^, Shakeel Ahmad^5^_,_ Sajjad Hussain^6^, Muhammad Arshad^7^, Summia Muneer^8^_,_ Aqib Hassan Ali Khan^9^, Shah Fahad^10^, Rahul Datta^11,^ Mazhar Iqbal^12^, Timothy D. Schwinghamer^2^

^1^Department of Soil Science, Faculty of Agricultural Sciences and Technology, Bahauddin Zakariya University, 60800 Multan, Pakistan

^2^Department of Plant Sciences, Faculty of Agricultural and Environmental Sciences, McGill University, Macdonald Campus 21111, Lakeshore Road, Ste-Anne-de-Bellevue, H9X 3V9 Quebec, Canada

^3^State Key Laboratory of Soil and Sustainable Agriculture, Institute of Soil Science, Chinese Academy of Sciences, 210008 Nanjing, China

^4^Department of Plant Pathology, Faculty of Agricultural Sciences and Technology, Bahauddin Zakariya University, 60800 Multan, Pakistan

^5^Department of Agronomy, Faculty of Agricultural Sciences and Technology, Bahauddin Zakariya University, 60800 Multan

^6^Department of Horticulture, Faculty of Agricultural Sciences and Technology, Bahauddin Zakariya University, 60800 Multan, Pakistan

^7^Institute of Environmental Sciences and Engineering, School of Civil and Environmental Engineering, National University of Sciences and Technology (NUST), 44000 Islamabad, Pakistan. Pakistan.

^8^Institute of Plant Breeding and Biotechnology, Muhammad Nawaz Sharif, University of Agriculture, Multan, Pakistan

^9^Department of Earth and Environmental Sciences, Bahria University, Karachi Campus, Karachi, 75260, Pakistan

^10^Department of Agronomy, The University of Haripur, Haripur 22620, Pakistan

^11^Department of Geology and Pedology, Faculty of Forestry and Wood Technology, Mendel University in Brno, Zemedelska1, 61300 Brno, Czech Republic

^12^Department of Environmental Sciences, Faculty of Biological Sciences, Quaid-i-Azam University, Islamabad 45320, Pakistan

***Correspondance:**

1 : Dr. Ghulam Mustafa Email : [ghulammustafa7944@yahoo.com](mailto:ghulammustafa7944@yahoo.com)

2 : Dr. M. Arif Ali. Email: [arif1056@bzu.edu.pk](mailto:arif1056@bzu.edu.pk)

3 : Dr. Niaz Ahmed Email : niaz.ahmad@bzu.edu.pk

**Running title:** Muskmelon and sanitation of composts

**Table S1.** %AFP of the media.

|  | **Proportions (% v/v) of particle size (mm)** | | | | | **%AFP of the composts** | | | | |
| --- | --- | --- | --- | --- | --- | --- | --- | --- | --- | --- |
| Treatments | <0.5 | 0.5-1 | 1-2 | 2-3.3 | 3.3-5.0 | Guar | Jantar | Wheat straw | Rice hull | Peat moss |
| Peat moss | 63 | 20 | 0 | 12.5 | 4.5 | 0 | 0 | 0 | 0 | 10.00 a |
| T1 | 50 | 40 | 10 |  |  | 4.9 i | 5.3 g | 5.2 hi | 4.6 f | 0 |
| T2 | 50 | 30 | 20 |  |  | 5.6 ghi | 6.3 efg | 5.1 i | 4.8 ef | 0 |
| T3 | 50 | 20 | 30 |  |  | 5.6 ghi | 5.7 fg | 5.3 ghi | 4.8 ef | 0 |
| T4 | 50 | 10 | 40 |  |  | 6.7 fgh | 6.7 ef | 6.3 fgh | 5.4 def | 0 |
| T5 | 50 | 0 | 50 |  |  | 7.2 def | 7.4 cde | 6.4 efg | 5.9 bcd | 0 |
| T6 | 0 | 50 | 50 |  |  | 9.0 abc | 9.4 ab | 9.7 ab | 6.3 abcd | 0 |
| T7 | 10 | 50 | 40 |  |  | 8.5 bcd | 6.5 bc | 8.4 cd | 6.5 abc | 0 |
| T8 | 20 | 50 | 30 |  |  | 6.9 ef | 7.2 de | 6.9 ef | 6.6 abc | 0 |
| T9 | 30 | 50 | 20 |  |  | 6.8 efg | 6.7 efg | 6.3 fgh | 6.3 abc | 0 |
| T10 | 40 | 50 | 10 |  |  | 6.2 fgh | 6.3 efg | 6.4 efg | 6.2 abcd | 0 |
| T11 | 50 | 50 | 0 |  |  | 5.6 hi | 5.6 fg | 5.7 ghi | 5.7 cde | 0 |
| T12 | 10 | 40 | 50 |  |  | 9.8 a | 10.1 a | 10.4 a | 6.5 abc | 0 |
| T13 | 20 | 30 | 50 |  |  | 9.2 ab | 9.3 ab | 9.3 bc | 6.9 a | 0 |
| T14 | 30 | 20 | 50 |  |  | 7.9 cde | 8.4 bcd | 8.4 cd | 6.7 ab | 0 |
| T15 | 40 | 10 | 50 |  |  | 7.1 def | 7.2 de | 7.5 de | 6.6 ab | 0 |

Small case letters exhibit significance at *p*≤ 0.05 level.

**Table S2.** %AFP of the media.

|  | | Proportions (%, v/v) of  particle size (mm) fractions | | %AFP | | | Proportions (%, v/v) of  particle size (mm) | | %AFP |
| --- | --- | --- | --- | --- | --- | --- | --- | --- | --- |
| Sr. No. | Treatments | < 2 | 2-3.3 | Guar | Jantar | Wheat straw | Rice hull  0.5-1 mm | 1-2 mm |  |
| 1 | T1 | 100 | 0 | 10.0 g | 10.2 h | 9.7 i | 0 | 100 | 10.7 a |
| 2 | T2 | 90 | 10 | 12.1 f | 12.1 g | 12.2 h | 10 | 90 | 10.2 ab |
| 3 | T3 | 80 | 20 | 14.1 e | 13.8 f | 14.0 g | 20 | 80 | 9.5 bc |
| 4 | T4 | 70 | 30 | 15.0 e | 15.0 f | 15.1 f | 30 | 70 | 8.5 cd |
| 5 | T5 | 60 | 40 | 18.8 d | 18.8 e | 18.6 e | 40 | 60 | 7.5 de |
| 6 | T6 | 50 | 50 | 20.0 b | 20.3 d | 20.4 d | 50 | 50 | 6.5 ef |
| 7 | T7 | 40 | 60 | 21.0 c | 20.8 d | 21.7 c | 60 | 40 | 6.5 ef |
| 8 | T8 | 30 | 70 | 21.5 b | 21.4 cd | 20.9 d | 70 | 30 | 6.3 f |
| 9 | T9 | 20 | 80 | 21.8 b | 23.9 a | 21.8 bc | 80 | 20 | 6.0 fg |
| 10 | T10 | 10 | 90 | 23.5 a | 22.0 b | 22.5 b | 90 | 10 | 6.5 fg |
| 11 | T11 | 0 | 100 | 24.1 a | 24.5 a | 23.5 a | 100 | 0 | 5.0 g |

All the values are means of three replicates of %AFP values, whereas small case letters exhibit significance at *p*≤ 0.05 level.

**Table S3.** Physico-chemical properties of the media.

| **Treatments** | **Water-holding capacity (%)** | **Shrinkage (%v/v)** | **Dry bulk density (g cm^-3^)** |
| --- | --- | --- | --- |
| **Acceptable ranges** | **55.00-80.00** | **< 30.00** | **≤ 0.40** |
| Control (Peat moss) | 54.00 d | 14.90 a | 0.18 f |
| Guar | 74.41 ab | 13.70 bc | 0.41 b |
| Jantar | 78.13 a | 13.80 b | 0.38 cd |
| Wheat straw | 70.50 bc | 12.60 d | 0.33 e |
| Rice hull | 53.50 de | 13.43 bcd | 0.43 a |

Rice hull, jantar, wheat straw and jantar composts were of 10% AFP. All the values are means of three replicates, whereas letters exhibit significant differences among the treatments at *p*≤ 0.05 level.
